# 387. Predictors of Seropositivity to SARS-CoV-2 Among Workforce Members at a Large Urban Medical Center

**DOI:** 10.1093/ofid/ofab466.588

**Published:** 2021-12-04

**Authors:** Evelyn A Flores, Deborah Kupferwasser, Prudencio Merino, Donna Phan Tran, Honghu Liu, Yilan Huang, Michael Bolaris, Megan H Nguyen, Mildred Gonzalez, Wellington Da Silva, Leslie Astorga-Cook, Angel Abueg, Holli Mason, Loren G Miller

**Affiliations:** 1 Division of Infectious Diseases, the Lundquist Institute at Harbor-UCLA Medical Center, Torrance, CA, Torrance, California; 2 University of California, Los Angeles, School of Dentistry, Public Health and Medicine, Los Angeles CA, Los Angeles, California; 3 University of California, Los Angeles, Department of Biostatistics, Los Angeles CA, Los Angeles, California; 4 University of California Irvine, Irvine, California; 5 Los Angeles County College of Nursing and Allied Health, Los Angeles, CA, Los Angeles, California; 6 Los Angeles County+ USC Medical Center, Los Angeles, CA, Los Angeles, California; 7 Department of Pathology, Harbor-UCLA Medical Center, Torrance, CA, Torrance, California; 8 Harbor UCLA, Torrance, California

## Abstract

**Background:**

Prior to SARS-CoV-2 vaccination availability, medical centers workers were at significant COVID-19 (COVID) infection risk. As part of a program offering free SARS-CoV-2 serology tests to medical center employees, we examined risk factors for prior COVID infection.

**Methods:**

From Sept. to Dec. 2020, we advertised free IgG antibody testing to all Los Angeles County-Univ. of Southern California Medical Center (LAC+USC) workforce members (clinical and non-clinical) via repeated email blasts. Antibody was determined using the Abbott SARS-Cov-2 IgG test against SARS-CoV-2 nucleocapsid protein. Program participants were asked to fill out a detailed epidemiologic questionnaire about work and non-work COVID risks on their cell phone or on paper at the time of phlebotomy. All testing was done prior to COVID vaccine availability.

**Results:**

Among approximately 10,500 workforce members, 1327 had serologies done. Among those 1273 (96%) completed the questionnaire and were included in the analysis. SARS-CoV-2 IgG antibodies were found in 60 (4.7%). In bivariate analysis, we found associations between SARS-CoV-2 seropositivity and persons who previously tested positive for COVID (OR 175.8 [95% CI 77.6 – 398.6]), persons who thought they had prior COVID but tested negative (OR 3.9 [95% CI 1.3 – 11.4]), and persons who thought they had prior COVID but did not get a COVID test (OR 4.2 [95% CI 1.4 – 12.5]). In a multivariate model of SARS-CoV-2 seropositivity examining work- and non-work-related COVID exposures (Table), seropositivity was associated with work-related COVID exposure without adequate personal protective equipment (PPE) (OR 5.1 [95% CI 2.1 – 12.2]), work-related COVID exposure with adequate PPE (OR 3.5 [95% CI 1.5 – 8.0]), never wearing a mask outside of work (OR 7.1 [95% CI 1.3 – 38.4]), and Native Hawaiian/Pacific Islander race (OR 6.6 [95% CI 1.7 – 23.4]). Seropositivity was inversely associated with living at home with multiple age groups (OR 0.4 [95% CI 0.2 – 0.8]). Multivariate Model of Exposures Associated with Positive COVID Serology Among LAC+USC Workforce Members

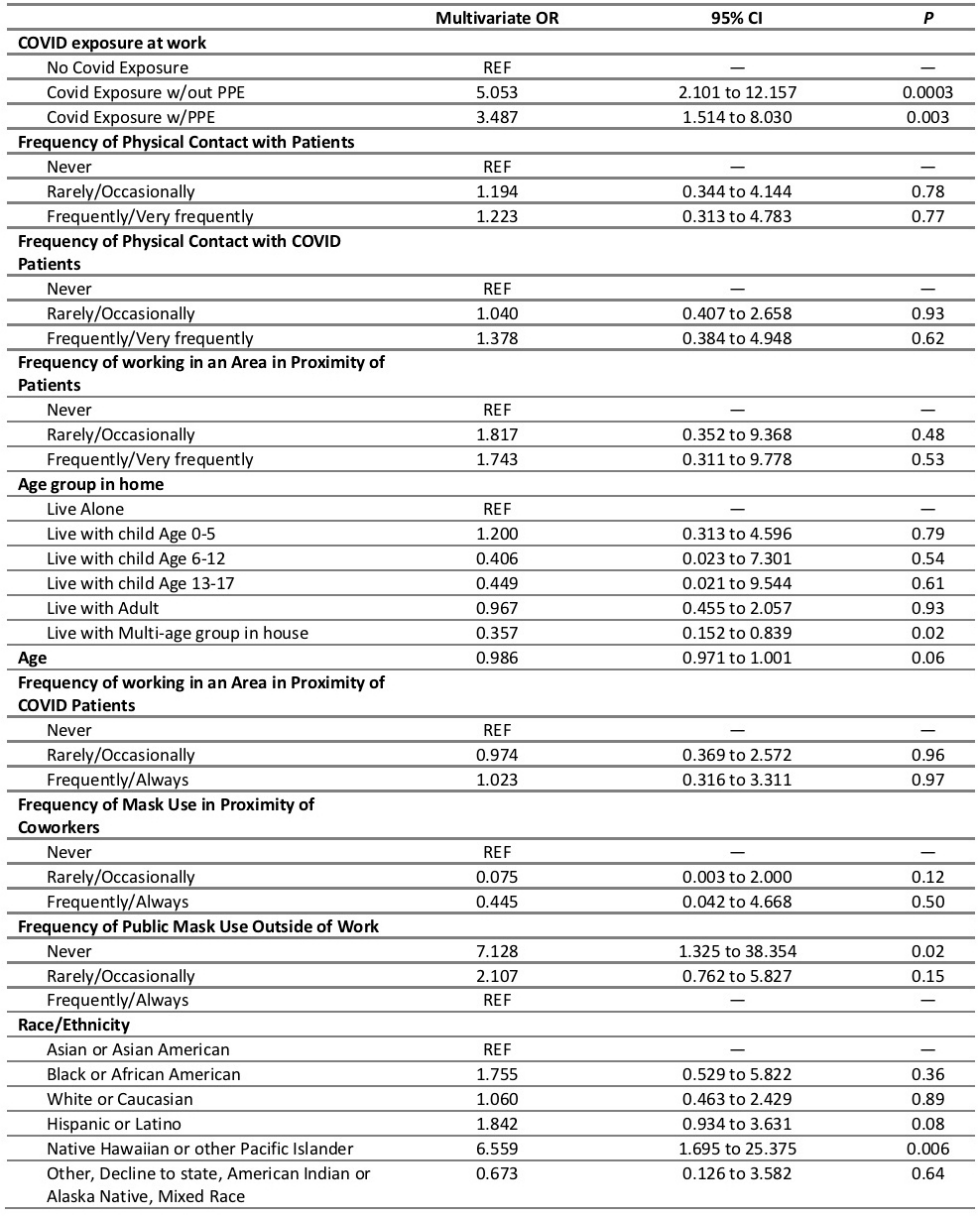

**Conclusion:**

Among workers in a large urban medical center prior to COVID vaccine availability, SARS-CoV-2 seropositivity was associated with work-related COVID exposures and low mask use outside of work, suggesting that COVID transmission in workforce members occurs both via occupational and non-occupational routes.

**Disclosures:**

**Loren G. Miller, MD, MPH**, **Medline** (Grant/Research Support, Other Financial or Material Support, Contributed product)**Stryker** (Other Financial or Material Support, Contributed product)**Xttrium** (Other Financial or Material Support, Contributed product)

